# Lipid emulsion mitigates impaired pulmonary function induced by limb ischemia/reperfusion in rats through attenuation of local cellular injury and the subsequent systemic inflammatory

**DOI:** 10.1186/s12871-017-0375-6

**Published:** 2017-06-19

**Authors:** Fangfang Xia, Yun Xia, Sisi Chen, Lulu Chen, Weijuan Zhu, Yuanqing Chen, Thomas J. Papadimos, Xuzhong Xu, Le Liu

**Affiliations:** Department of Anesthesiology, the First Affiliated Hospital of Wenzhou Medical University, Zhejiang, China

**Keywords:** Limb, Ischemia/reperfusion injury, Lung injury, Lipid emulsion, MDA

## Abstract

**Background:**

Limb ischemia/reperfusion causes inflammation and elicits oxidative stress that may lead to local tissue damage and remote organ such as lung injury. This study investigates pulmonary function after limb ischemia/reperfusion and the protective effect of a lipid emulsion (Intralipid).

**Methods:**

Twenty-four rats were divided into three groups: sham operation group (group S), ischemia/reperfusion group (group IR), and lipid emulsion treatment group (group LE). limb ischemia/reperfusion was induced through occlusion of the infrarenal abdominal aorta for 3 h. The microvascular clamp was removed carefully and reperfusion was provided for 3 h.

**Results:**

The mean arterial pressure in group LE was higher than group IR during the reperfusion period (*P* = 0.024). The heart rate of both group LE and IR are significantly higher than group S during the ischemia period(*P* < 0.001, *P* < 0.001, respectively). The arterial oxygen pressure of group LE was significantly higher than group IR (*P* = 0.003), the arterial carbon dioxide pressure of group LE were lower than that of group IR (*P* = 0.005). The concentration of plasma interleukin-6, tumor necrosis factor-α and malondialdehyde in group LE were significantly lower than group IR (*P* < 0.001, *P* = 0.009 and 0.029, respectively). The plasma superoxide dismutase activity in group LE was significantly higher than group IR (*P* = 0.029). The myeloperoxidase activity in lung tissues of group LE was significantly less than group IR (*P* = 0.046). Both muscle and lung in group IR were damaged seriously, whereas lipid emulsion (Intralipid) effectively reversed the damage. In summary, Intralipid administration resulted in several beneficial effects as compared to group IR, such as the pulmonary gas exchange and inflammatory.

**Conclusions:**

The ischemic/reperfusion injury of limb muscles with resultant inflammatory damage to lung tissue can be mitigated by administration of a lipid emulsion (Intralipid, 20%, 5 ml/kg). The mechanisms attenuating such a physiological may be attributed to reduction of the degree of limb injury through a decrease in the release of local inflammatory mediators, a reduction of lipid peroxidation, and a blunting of the subsequent remote inflammatory response.

## Background

Acute limb ischemia is a frequent clinical entity that is associated with trauma, acute atherosclerotic thrombosis/embolism or iatrogenic vascular injuries [1]. The most common ischemic/reperfusion injury in surgery are vascular clamps for vascular surgery as well as Tourniquets, used for orthopedic surgery of the limbs. Reperfusion of the acutely ischemic limb may cause significant local tissue damage and release of toxic metabolites [2] that initiate a cascade of events that may result in single or multiple organ dysfunction [3, 4].

A variety of animal studies have demonstrated the lung is the primary affected organ secondary to inflammatory mediators after limb ischemia/reperfusion. The local release of such mediators after local injury lead to secondary increases in pulmonary vascular permeability and neutrophil sequestration, which may result in gas exchange impairment [5] and acute respiratory distress syndrome [6, 7]. Yassin et al. [2] reported increased tumor necrosis factor-α (TNF-α) and interleukin-6 (IL-6) levels are associated with vascular obstruction, and activation and infiltration of neutrophils in the lung after limb ischemia/reperfusion. Shih et al. [1] have suggested that TNF-α, IL-1β, IL-6 in limb tissues after limb ischemia/reperfusion lead to the activation of polymorphonuclear leukocytes (PMNs) and increase the expression of cell adhesion molecules (CAMs) on both neutrophils and endothelial cells, resulting in local tissue injury and downstream damage to the lungs. However, investigators have also proved that glutamine can mitigate local injury thereby inhibiting a systemic inflammatory affect. Somuncu et al. [8] postulated that Trapidil prevents secondary lung injury induced by IR through its inhibitory action on PMN activation and reducing the levels of malondialdehyde (MDA). Additionally, Wang et al. [5] reported that Shenmai can improve secondary lung dysfunction after limb ischemia/reperfusion by attenuating inflammatory and oxidative response.

Intralipid, as a long chain triglyceride, is the first lipid emulsion safely used in humans. Intralipid was shown to reduce the infarct size in the ex vivo model of IR in rats [9]. Rahman et al. [10] manifested that Intralipid also has been proposed as a powerful agent for cardioprotection against ischemia/reperfusion by activating GSK-3β to restrain mPTP via PI3K-Akt/ERK pathways. Furthermore, lipid emulsions have been a rescue therapy for local anesthetic-induced cardiotoxicity [11]. Moreover, lipid emulsion was shown to possess an anti-inflammatory capacity through its inhibition of IL-1β and TNF-α production in vitro [12]. However, there is no study as of yet have reported the mitigating affect of lipid emulsions on the production of local toxic metabolites and their resulting effect on the lungs after limb ischemia/reperfusion.

We hypothesize that lipid emulsion administration immediately before reperfusion after the limb ischemia attenuates the inflammatory effects of limb ischemia/reperfusion thereby protecting limb tissue from local injury that may lead to remote organ damage. Therefore, we established an limb ischemia/reperfusion model in rats to observe whether lipid emulsion can attenuate the lung injury after limb ischemia/reperfusion.

## Methods

### Experimental animals

All animal protocols were approved by the Wenzhou Medical University Animal Care and Use Committee (wydw2013–0023, Wenzhou, China). The care and handling of the animals were in accordance with National Institutes of Health guidelines. Adult male SD rats weighing 300–350 g were obtained from Shanghai Slac Laboratory Animal Co., Ltd. (Shanghai, China) and housed in plastic cages (4 rats per cage) at the animal center of Wenzhou Medical College with a 12 h day/night cycle at an ambient temperature of 19–25 °C and at 55–60% humidity. All rats were fed a standard laboratory nutritional regimen and were provided water ad libitum until the morning of experiment.

### Experimental protocol (lower limb ischemia/reperfusion protocol)

The rats were fasted for 12 h before the experiments with ad libitum access to water. On the day of the experiment, rats were anesthetized with chloral hydrate (5%, 350 mg/kg). The left external jugular vein (for intravenous injection) and the right carotid artery (for continuous blood pressure monitoring and blood collection) were cannulated with polyethylene (PE-22G, 0.9 mm diameter) catheters. After completion of invasive procedures, the rats were kept in the supine position for the duration of the experiment and allowed to breath spontaneously. Heparin (50 unit) was administered intravenously to avoid the formation of thrombosis and then the rats were allowed to stabilize for 30 min. To achieve bilateral lower limb ischemia/reperfusion, the infrarenal abdominal artery was occluded by microvascular clamps (length, 1.4 cm) followed by 3 h of ischemia of the hind limbs. Thereafter, the microvasuclar clamp was released and the lower limbs were reperfused for 3 h. Hydration of the animals was maintained by injection of sterile 0.9% sodium chloride solution 3 ml·kg^−1^·h^−1^subcutanesously. Body temperature was maintained at 37 ± 0.5 °C with a heating lamp held at a safe distance. Electrocardiography, using three subcutaneous needle electrodes, and the arterial pressure were recorded continuously throughout the duration of the protocol by a MedLab data archiving and retrieval system using U/4C051 (Nanjing Medease Science and Technology Co., Ltd., Jiangsu, China). The mean arterial pressure (MAP) and heart rate (HR) were continuously monitored and recorded once an hour until the end of the experiments. All animals were euthanized at the end of the 3 h of reperfusion, then the lung tissue and plasma samples were obtained to determine pulmonary pathology, assay lung wet/dry (W/D) ratio, PaO_2_, PMN count, measurement of the plasma content of IL-6, TNF-α, superoxide dismutase (SOD) activity and MDA levels; and myeloperoxidase (MPO), SOD activity, and MDA content in the lung tissue.

### Experimental protocols

Twenty-four adult male SD rats weighing from 300 to 350 g, were randomly divided into 3 groups (*n* = 8): (1) sham operation group (group S): the infrarenal abdominal artery were exposed but not occluded; (2) ischemia reperfusion group (group IR): 5 ml/kg of saline before reperfusion was administered immediately after reperfusion; (3) lipid emulsion treatment group (group LE): infusion with a bolus of LE (20% Intralipid, 5 ml/kg, Wuxi huarui pharmaceutical co., LTD., NO. 80BE069) just after reperfusion.

The dosage of Intralipid and the time to administration in this study were selected to match the dosages and the times used in previous studies regarding the treatment of the heart IR injury [10] and the lung injury induced by limb ischemia/reperfusion [11].

### Arterial blood gas (ABG) and determination of plasma mediators levels

In this study, pulmonary function was assessed using arterial blood gas (ABG) analyses and clinical outcomes. Immediately before induction of ischemia, an arterial blood (0.5 ml) was drawn and ABG levels were measured immediately with an i-STAT Portable Clinical Analyzer (i-STAT Corporation, east Windsor, NJ). At the end of 3 h of reperfusion (before euthanization), another ABG was drawn. The remaining blood sample (4 ml) taken before euthanization was centrifuged (4000 rpm at 4 °C for 10 min) to separate the plasma, which was then stored at −80 °C for subsequent analysis of IL-6, TNF-α, MDA and SOD. IL-6, TNF-α were determined using commercially available enzyme-linked immunosorbent assay kits (Westang biotechnology Co. Ltd., Shanghai, China), and the plasma MDA and SOD were analyzed using the thiobarbituric acid reaction and the xanthine oxidase reaction methods (Jiancheng Bioengineering Research Institute, Nanjing, China) according to manufacturer’s instructions. The determination of these levels was performed by investigators who were blinded to the experiment (and blood gas results).

### Tissues collection and lung wet to dry weight (W/D) ratio

All rats were euthanized after 3 h of ischemia and 3 h of reperfusion, then the main bronchi and two lungs were removed. After dividing the upper and lower lobes of left lung, the left upper lobe lung tissues and right limb muscle were infused with 4% formaldehyde; the left lower lobe lung tissues were used for subsequent W/D weight ratio assay. The right lung tissues were snap frozen in liquid nitrogen and stored at −80 °C for subsequent analysis. In order to determine lung water content (W/D weight ratio assay), the freshly harvested left lower lobe was weighed and then placed in the oven for 48 h at 80 °C and weighed again when it was dry. The W/D weight ratio was then calculated.

### Malondialdehyde (MDA) assay and superoxide dismutase (SOD) assay for lipid peroxidation status evaluation

MDA levels reflect the peroxidation rate of lipids in tissue. In brief, snap-frozen lung tissues were thawed and tissues homogenates were collected. Then, phosphoric acid and thiobarbituric acid solution were added to homogenates (0.05 mL) of the plasma sample (0.05 mL) and the mixture was heated in boiling water (40 min). After cooling and centrifugation at 3500 rpm for 10 min, the absorbance of the organic layer was measured at 532 nm to determine the amounts of lipid peroxides. MDA levels were expressed as nmol/mg protein in tissues and nmol/ml in plasma.

SOD activity was measured by the superoxide radicals through xanthine and xanthine oxidase reaction, the radicals can react with p-iodonitrotetrazlium violet (INT) to form a red formazan dye, which can be measured at 550 nm. Assay media were added to homogenates (0.05 mL) and the plasma sample (0.05 m); the mixture was heated by the water bath at 37 °C for 40 min. They were then placed at room temperature for 10 min, and the absorbance of the organic layer was measured at 550 nm. SOD activity was expressed as U/mg protein in tissues and U /ml in plasma.

### Myeloperoxidase (MPO) activity assay

Lung MPO activity was also quantified in order to measure lung injury. Snap frozen tissue samples (−80 °C) were homogenized, resuspended, sonicated, and centrifuged. After separation, the supernatant was incubated in a water bath for 2 h at 60 °C and MPO activity was then assessed by measuring the H_2_O_2_-dependent oxidation of O-dianisidine using a spectrophotometry (UV-VIS 8500, TianMei Co. Ltd., Shanghai, China) at 460 nm. The results were expressed as U/g wet tissue weight.

### Histologic analysis, muscle injury score (MIS), lung injury score (LIS) and PMNs counting and HE staining

The formaldehyde-infused right limb tissues and left upper lobe lungs were stained with hematoxylin and eosin after embedding them in paraffin wax and then serially sectioned. The limb tissues (HPF, ×100) and the lungs (HPF, ×200) were observed under the microscope (Olympus BX-51, Japan).

According to Hori et al. [13], the damage score of muscle tissue sections was assigned based on the following scales: 1 = disorganization and degeneration of the muscle fibers (0, normal; 1, mild; 2, moderate; 3, severe) and 2 = inflammatory cell infiltration (0, normal; 1, mild; 2, moderate; 3, severe). Summation of the two scores of muscle tissue determined the muscle injury score (MIS) (range, 0–6). The scoring system used to assess the degree of lung injury was from Kao et al. [14], with some modifications based on the following histologic features: edematous changes of the alveolar wall, hemorrhage, vascular congestion, and PMN infiltration. Each histologic characteristic was scored (0-normal to 5-severe), and overall lung injury was then categorized according to the sum of the score. The score of lung injury (LIS) was compared to the average of the sum of each field score. Evaluation of histologic characteristics and scoring of muscle and lung injury was performed by a pathologist, who was blinded to the grouping.

The degree of leukocyte infiltration was assayed by determining PMN/alveoli ratio. In brief, PMNs and alveoli per high-power field (HPF, ×400) in 10 randomly selected areas of each sample were counted. The PMNs/alveoli ratio was determined by dividing the sum of the PMNs in 10 HPFs by that of the alveoli.

### Ultrastructure

Right limb tissues and left upper lobe lung tissues were cut into small pieces and immersed in universal fixative (1% glutaraldehyde, 4% paraformaldehyde, pH 7.4) immediately, then post-fixed in 2% osmium tetroxide, dehydrated in graded acetones, and embedded in an Epon-Araldite mixture (Fisher Scientific Co. Toronto, Canada). Thin-sections were made and mounted on copper grids; uranyl acetate and lead citrate were used for contrasting. The grids were examined for pneumocytes by electron microscopy (Hitachi, H-7500, Japan) (HPF, ×12,000).

### Statistical analysis

The determination of the number of animals in each groups was based on our preliminary study (*n* = 5), in which the PaO_2_ of arterial blood at the end of experiment were 95.00 ± 3.64、76.80 ± 8.26 and 91.20 ± 7.43 in group S, group IR and group LE, respectively. Using a two-tailed type one error at 5% and type two error of 10% (α = 0.05, β = 0.1), the sample size of 7 per group was obtained by Power Analysis and Sample Size (PASS; 11.0). To account for potential attrition, we enrolled 8 rats per group.

SPSS (version 15.0; SPSS Inc., Chicago, IL) was used to carry out the computations. Data were tested for normal distribution using the Shapiro–Wilk test. Continuous, normally distributed variables were presented as mean ± standard errors or medians and interquartile values. Values for baseline parameters, lung W/D weight ratio, ABG values except PaCO_2_, SaO_2_, lactate, PMN counts, the concentrations of various indexes in lung tissue and plasma, and LIS were compared by one-way ANOVA. For homogeneity of variance data, we used the LSD test, other heterogeneity of variance data were tested using Brown-Forsythe and the Dunnett’T3 test. Continuous hemodynamic variables (MAP and HR) in animals were compared across time by two-way repeated measures ANOVA, with Bonferroni correction post-testing when significance was achieved (*P* < 0.05). Non-normal distributed data PaCO_2_, SaO_2_, lactate and MIS among the groups were both compared using an initial Kruskal–Wallis H-test and then the Mann-Whitney U test was used between two groups when significance was achieved (*P* < 0.05). Statistical significance was considered as *P* < 0.05.

## Results

Baseline values of weight, hemodynamic parameters, and ABG among these three groups were showed in Table 1. All the rats survived until the end of the experiment with stable hemodynamics.

**Table 1:** Baseline Vallues of Weight, Hemodynamic, and Blood Gas Values (*n* = 8 in Each Group)

	S	IR	LE
Weight (g)	316 ± 21	321 ± 11	322 ± 9
MAP (mmHg)	109 ± 11	110 ± 11	111 ± 10
HR (Beat/Min)	326 ± 16	331 ± 21	330 ± 21
Blood Gas Values			
pH	7.39 ± 0.03	7.36 ± 0.05	7.34 ± 0.03
PaCO_2_ (mmHg)	40.5 ± 5.7	43.0 ± 3.6	45.3 ± 5.5
PaO_2_ (mmHg)	105.4 ± 7.6	103.1 ± 6.4	102.8 ± 7.6
BE (mmol/L)	−0.6 ± 1.8	−0.9 ± 1.6	0.0 ± 1.6
HCO_3_^−^(mmol/L)	22.7 ± 3.0	23.6 ± 2.1	25.2 ± 3.0
SaO_2_ (%)	98 (97,98)	97.5 (97,98)	97 (97,99)
Lactate (mmol/L)	1.3 ± 0.4	1.6 ± 0.9	2.1 ± 0.7

Normally distributed data were given as mean ± SD, whereas non-normal distributed data were expressed as median and interquartile values. Baseline values for major parameters showed no significant differences among the three groups

### Hemodynamics measures

**Fig. 1:**
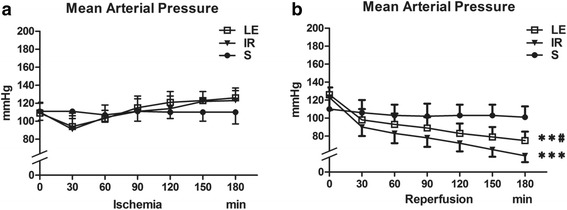
Mean arterial pressure (*n* = 8 in Each Group). **a** The MAPs showed no difference among three groups throughout the ischemia period. **b** The MAPs during the reperfusion period. Three groups displayed differences (*P* < 0.001). MAP in three groups were presented graphically. Data were given as mean ± SD. ** *p* < 0.01 (vs. S), *** *p* < 0.001 (vs. S). # *p* < 0.05 (vs. IR)

As shown in Fig. 1, the MAPs showed no difference among group S, IR and LE throughout the ischemia period (*P* = 0.812). However, they were different during the reperfusion period. The MAP of group IR was decreased significantly as compared to that of group S(*P* < 0.001), the MAP of group LE was increased significantly as compared to group IR(*P* = 0.024), but still lower than group S (*P* = 0.004).

**Fig. 2:**
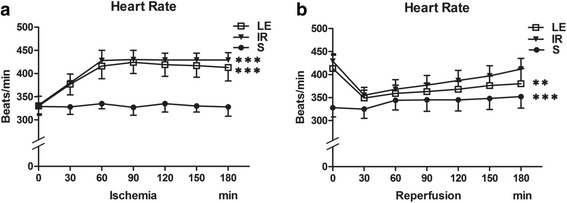
Heart rate (*n* = 8 in Each Group). **a** The HRs were different among three groups throughout the ischemia period(*P* < 0.001). **b** The HRs during the reperfusion period. Three groups displayed differences (*P* < 0.001). HR in three groups were presented graphically. Data were given as mean ± SD. ** *p* < 0.01 (vs. S), *** *p* < 0.001 (vs. S)

As shown in Fig. 2, the HRs showed no difference between group LE and IR throughout the ischemia period(*P* = 0.241), the HR of both group LE and IR are significantly higher than group S during the ischemia period(*P* < 0.001, *P* < 0.001, respectively). Moreover, they were different during the reperfusion period. The HR of group LE and IR was decreased significantly as compared to that of group S(*P* = 0.003, *P* < 0.001, respectively), while the HR of group LE showed no difference with group IR (*P* = 0.063).

### ABG Analysis

The final PaO_2_ and lactate of group IR were significantly lower than those of group S (*P* < 0.001 and *P* = 0.001, respectively), whereas the final PaCO_2_ of group IR was significantly higher than group S (*P* = 0.005; Table 2). The final PaO_2_, BE,HCO_3_, SaO_2_ and lactate of group LE were significantly higher than those of group IR (*P* = 0.003, 0.010, 0.044, 0.011 and 0.037, respectively), whereas end PaCO_2_ of group LE was significantly lower than group S(*P* = 0.005; Table 2). In contrast, the final ABG between the group LE and group S were not significantly different except HCO_3_. The final HCO_3_ was significantly higher in group LE than group S (*P* = 0.023; Table 2).

**Table 2:** Arterial Blood Gas Parameters, Wet-To-Dry Lung Weight Ratio, MIS, LIS and PMNs Counting at the end(*n* = 8 in Each Group)

	S	IR	LE
pH	7.39 ± 0.18	7.33 ± 0.67	7.37 ± 0.40
PaCO_2_ (mmHg)	48 (41,46)	58 (49,61)**	45 (43,46) ^##^
PaO_2_ (mmHg)	94 ± 5	79 ± 8***	90 ± 7^##^
BE (mmol/L)	0.9 ± 1.6	−1.3 ± 3.0	2.3 ± 2.7^#^
HCO_3_^−^(mmol/L)	24 ± 3	25 ± 3	27 ± 2*^#^
SaO_2_ (%)	95 (95,96)	94.5 (92,95)	96 (95,97) ^#^
Lactate (mmol/L)	0.6 ± 0.2	1.4 ± 0.7**	1.0 ± 0.3^#^
W/D	5.1 ± 0.2	5.6 ± 0.4	5.1 ± 0.3
MIS	0.5 (0.0,1.0)	4.5 (4.0,5.0)	3.0 (2.0,4.0)
LIS	3.1 ± 2.8	13.8 ± 3.6	6.9 ± 3.1
PMNs	21.0 ± 10.1	70.1 ± 16.4	31.0 ± 9.3

Normally distributed data were given as mean ± SD, whereas non-normal distributed data were expressed as median and interquartile values

* *p* < 0.05 (vs. S), ** *p* < 0.01 (vs. S), *** *p* < 0.001 (vs. S)

# *p* < 0.05 (vs. IR), ## *p* < 0.01 (vs. IR)

### Concentrations of plasma IL-6, TNF-α, SOD activity and MDA

**Fig. 3:**
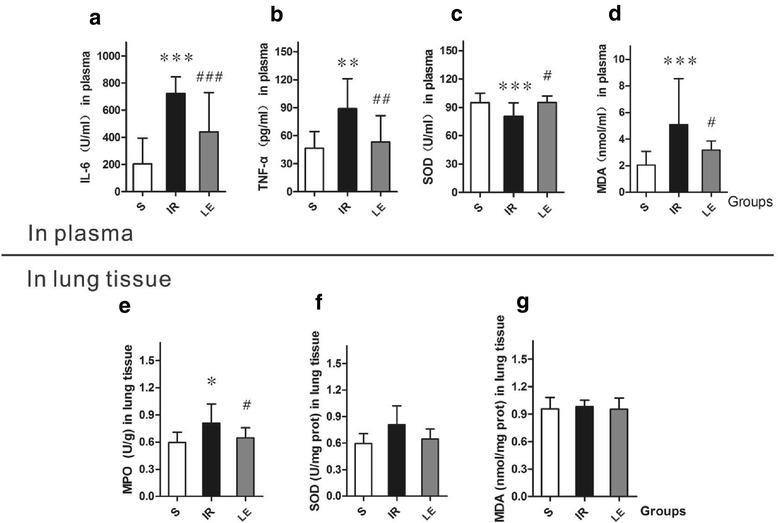
Concentration of IL-6, TNF-α, SOD activity and MDA in plasma and MPO, SOD activity and DA levels in lung tissues(*n* = 8 in Each Group). IL-6, interleukin-6; TNF-α, tumor necrosis factor-α; SOD, superoxide dismutase; MDA, malondialdehyde. MPO, Myeloperoxidase. Data were given as mean ± SD. * *p* < 0.05 (vs. S), ** *p* < 0.01 (vs. S), *** *p* < 0.001 (vs. S). # *p* < 0.05 (vs. IR), ## *p* < 0.01 (vs. IR), ### *p* < 0.001 (vs. IR). **a** Concentration of IL-6 in plasma displayed differences in the three groups (*P* = 0.001). **b** Concentration of TNF-α in plasma displayed differences in the three groups (*P* < 0.001). **c** Concentration of SOD activity in plasma displayed differences in the three groups (*P* =. **d** Concentration of MDA in plasma displayed differences in the three groups (*P* = 0.009). **e** Concentration of MPO Activity in lung tissues displayed differences in the three groups (*P* = 0.028). **f** Concentration of SOD Activity in lung tissues displayed no differences among the three groups (*P* = 0.089). **g** Concentration of MDA in lung tissues displayed no differences among the three groups (*P* = 0.170)

Group IR had higher IL-6, TNF-α, MDA levels and lower SOD activity levels in plasma than group S (*P* = 0.001, 0.092, 0.063, 0.467 respectively; Fig. 3a–d). Concentrations of IL-6, TNF-α, MDA in group LE were significantly lower than group IR after the treatment of lipid emulsion (*P* = 0.005, 0.008, 0.029 respectively; Fig. 3a–d), and SOD activity levels in group LE were significantly higher than that of group IR (*P* = 0.006, Fig. 3c). Concentrations of IL-6 in group LE were higher than group S (*P* < 0.001, Fig. 3a). Group LE and group S presented no statistically significant differences in TNF-α, MDA and SOD activity levels (*P* = 0.630, 0.984, 0.208 respectively; Fig. 3b–d).

### Histology and muscle injury score

**Fig. 4:**
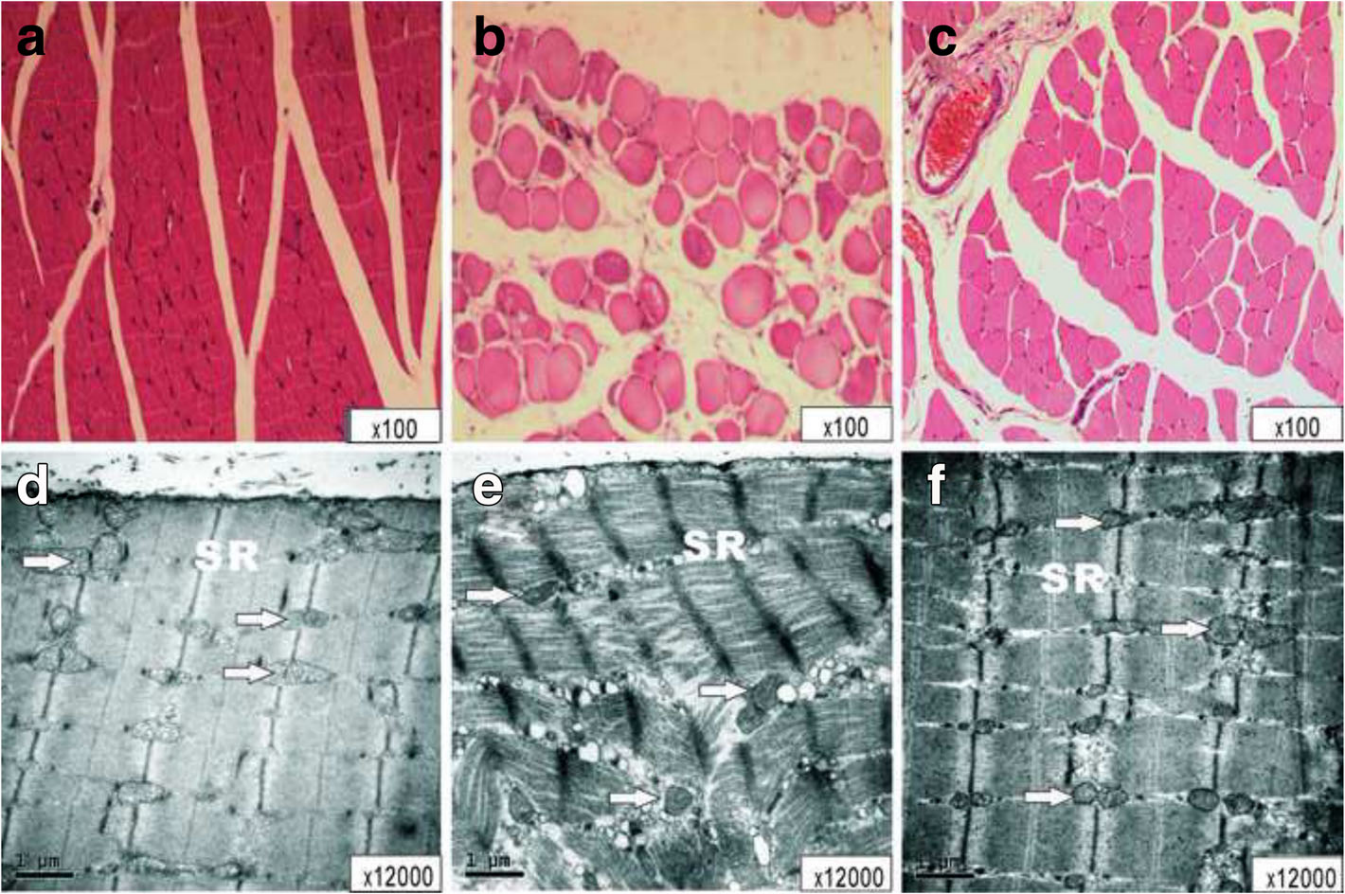
The light microscopy view of the right limb muscle in the S(A), IR(B), LE(C) groups, and the electron microscopy view of the right limb muscle in the S(D), IR(E), LE(F) groups. N, nucleus; SR, Sarcoplasmic reticulum; Mit, mitochondria; Lb, lamellar body. **a**, Muscle fibers were characterized to be normal, as having well-defined borders, consistent texture, and uniformity throughout the muscle fiber. **b**, IR group exhibited destruction of muscle fibers with edema and inflammatory cell infiltration. **c**, Near-normal muscle fibers are identified. **d**, Uninjured muscle fibers ultrastructural architecture were characterized as having well-defined borders, consistent texture, and uniformity throughout the muscle fibers. **e**, the muscles exhibited destruction with edma and inflammatory cell infiltration. Disorganization and fracure of muscle fibers, swelling of Lb, Mit and SR were observed severely. **f**, Incomplete and destructed of nucleus, Lb and Mit with swelling structures, but a much lower degree of damage than **e**

The light microscopy view of the right limb muscle fibers as shown in group S were characterized as normal; having well-defined borders, consistent texture, and uniformity. Histologic examination of muscle injury revealed evidence of skeletal muscle damage after 3 h of ischemia and 3 h of reperfusion. The muscles in group IR exhibited destruction of muscle fibers with accompanying edema and inflammatory cell infiltration (MIS in group IR vs. group S, *P* = 0.01). The MIS in group LE decreased significantly when compared to group IR (*P* < 0.001), but were still higher than group S (*P* < 0.001). The electron microscopy view of the muscle fibers as shown in group S were normal as exhibted by the uninjured muscle fiber ultrastructural architecture. Disorganization and fracture of muscle fibers, with swelling of lamellar bodies, mitochondria, and the sarcoplasmic reticululm, was severe in group IR (A,B). Group LE had some of the same ultrastructural changes as in group IR, but to a much lesser degree (Fig. 4).

### Lung W/D

As shown in Table 2, the left lower lobe W/D ratios in group IR were higher than group S (*P* = 0.002). The W/D ratios decreased significantly in group LE in comparison with group IR (*P* = 0.006), but showed no differences with group S (*P* = 0.687, Table 2).

### Concentrations of lung MPO, SOD activity and MDA

The three groups displayed differences in MPO activity level in lung tissues (*P* = 0.028). MPO concentrations in group IR were higher than group LE and group S (*P* = 0.046; *P* = 0.011, respectively). Group LE and group S presented no differences in MPO (*P* = 0.514, Fig. 3e). There were no significant difference in SOD activity and MDA levels among three groups (*P* = 0.089, *P* = 0.170, respectively; Fig. 3f and g).

### Histology and lung injury score

**Fig. 5:**
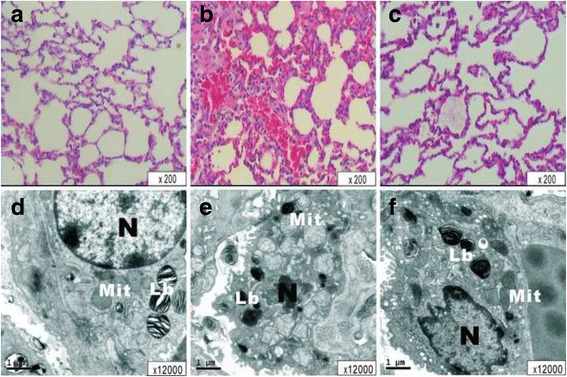
The light microscopy view of the left upper lobe lung in the S(A), IR(B), LE(C) groups, and the electron microscopy view of the left upper lobe lung in the S(D), IR(E), LE(F) groups. N, nucleus; Lb, Lamellar bodies; Mit, mitochondria. **a**, structure of alveoli are normal, there is no accumulation of inflammatory cells or erythrocytes observed in the alveloli. **b**, structure of alveoli are injured severity and widely, there were numerous inflammatory cells and erythrocytes associated with the edema and thickening in both alveolar wall and interstitial. **c**, microscopy examination of LE group revealed to be light damage in alveolar framework, inflammatory cells, erythrocytes and the situation of edema were alleviated visibly in LE group. **d**, in S group lung ultrastructural architecture was normal with type II pneumocytes containing normal Lb, no swelling of the mitochondria. **e**, Swelling of Lb and Mit in some type II pneumocytes were observed in group IR; Disintegrated naked nuclei and other structure of the amorphous necrotic material were observed in the alveolar cavity in IR group. **f**, similar incomplete and destructed of nucleus, Lb and Mit with swelling structures, but to a much lower degree than observed in LE group

The light microscopy view of the left upper lobe lung showed that the pulmonary architecture was normal in Group S. After IR injury, destruction of alveolar structures, neutrophil infiltration, thickening of the septal space, and intra-alveolar hemorrhage with debris were observed, as the lung injury scores (LIS) and PMN count in group IR were significantly higher than group S (*P* < 0.001, *P* < 0.001, respectively; Fig. 3). Group LE had lower LIS and PMN counts than group IR (*P* < 0.001, *P* < 0.001, respectively), but still higher than group S (*P* = 0.028, *P* = 0.019, respectively; Table 2). The electron microscopy view of the left upper lobe lung in group S was normal with type II epithelial cells containing normal lamellar bodies, no swelling of the mitochondria. However, in Group IR, there was swelling of lamellar bodies and mitochondria in a number of type II epithelial cells were observed in group IR. Disintegrated nuclei and other amorphous necrotic material were also observed in the alveolar cavity in Group IR. However, in group LE (Fig. 5) there was a lesser degree of nuclear damage and less cell edema in regard to lamellar bodies and mitochondria.

## Discussion

In this study, we demonstrated that Intralipid administered immediately after reperfusion, induced by bilateral lower limb ischemia/reperfusion, can mitigate acute lung injury in rats. We found that Intralipid administration resulted in several beneficial effects as compared to group IR. First, there was improvement of the pulmonary gas exchange as evidenced by significant increases in PaO_2_ and decreases in PaCO_2_. Second, there were decreases in IL-6, INF-α, and MDA concentrations, and increased SOD activity level in plasma. Third, the histologic findings showed that damage to the muscle and lung tissues were less severe after the administration of lipid emulsion. MIS, LIS and PMNs in lung tissues were all significantly lower than group IR, but still higher than group S. These results support the concept that a lipid emulsion acts through anti-inflammatory and/or anti-oxidative capacities to exert its protective effects against local limb muscle and acute lung injury caused by limb ischemia/reperfusion.

We have successfully established a modified limb ischemia/reperfusion and secondary lung injury model in rats by occluding the infrarenal abdominal artery for 3 h and allowing 3 h of reperfusion thereafter. Our model’s muscle injury, resultant impairment of pulmonary gas exchange, and systemic inflammation were in concordance with previous reports from Shih et al.^1^ and Yassin et al. [2].

Prolonged ischemia/reperfusion times in tissues can initiate direct damage of mitochondria and activate proteolytic enzymes. Jurmann et al. [15] found that fatty acids (the primary composition of lipid emulsions) can activate important bioactivity mediums by changing the integrity and mobility of cytomembrane, and may even impact cell signaling of apoptosis, inflammatory and immune responses. Yaqoob et al. [16] reported that fatty acid could combine with PPAR-α(Peroxisome proliferator-activated receptor alpha) directly, thereby influencing their attachment, and in doing so, affect the stabilization of lipid rafts, inhibiting immune response and decreasing the extent of local tissue injury. Lipid raft which was first mentioned by Simons et al. [17] which are dynamic microenvironments in the exoplasmic leaflets of the phospholipid bilayer of plasma membranes and are thought preferentially to group transmembrane proteins according to their function. Accordingly, the histologic examination of muscle injury in this study revealed that lipid emulsion administration attenuates muscle damage following limb ischemia/reperfusion, as demonstrated by less tissue destruction, less structural edema, and increased numbers of mitochondria, and a much smaller degree of overall damage than in group IR. These results indicate that administration of a lipid emulsion attenuates local limb injury by preventing/minimizing cellular damage and the resultant inflammatory reaction (local and systemic).

Shih et al. [1] reported that an excessive production of inflammatory cytokines (TNF-α, IL-1β, IL-6) may lead to the activation of neutrophils and the upregulation of CAMs on both neutrophils and endothelial cells. This is followed by transmigration into the local tissue interstitium resulting in local tissue damage, as well as an intense inflammatory responses that occur both locally and systemically [18]. Our results are in agreement with Kao et al. [14], who proved significant reductions of PaO_2_ and elevations of PaCO_2_ after reperfusion in rats undergoing lower limb ischemia/reperfusion. This can result in pulmonary edema [19] induced by the outflow of osmotic proteins from the microvascular endothelium, and a cascade amplification of the inflammatory response [20], which is followed by injury to the pulmonary vascular endothelium and pulmonary epithelium. The significantly high levels of IL-6, TNF-α, MDA, and the increase of PMNs in lung tissues in group IR support the notion that the systemic inflammatory response and lipid peroxidation may be involved in mechanisms concerning pulmonary dysfunction after limb ischemia/reperfusion. Although from this data it is not possible to confirm the source of the cytokines and MDA observed during our study; local/peripheral injury to the limbs is the most likely source. Reimund et al. [12] documented that lipid emulsion can inhibit of IL-1β and TNF-α production in vitro. Our study provided the first evidence demonstrating that lipid emulsion administration immediately after reperfusion mitigates acute lung injury in rats experiencing bilateral lower limb ischemia/reperfusion.

Our data also strongly indicates that lipid emulsion administration immediately after reperfusion can improve the ability of SOD in eliminating oxygen free radicals, and reducing MDA level in plasma. These results demonstrate that lipid emulsion significantly inhibits MDA elevations, indicating that it can provide a protection for lung injury after limb ischemia/reperfusion. However the levels of SOD and MDA in lung tissue showed no significant difference among the three groups after limb ischemia/reperfusion in rats. This suggests that the impairment of pulmonary gas exchange was not independent of oxidative stress injury and that lipid emulsion administration reduced lipid peroxidation in muscle tissues. Our results are in agreement with somef reports [1, 8], but contrary to the reports of others [5, 14]. The reasons for the discordance between these two kinds of different reports may be related to animal species, the duration of ischemia and/or reperfusion. The role of lipid emulsion in lipid peroxidation is not yet well defined and needs further examination.

The “Lipid sink” theory was first proposed in the study in which lipid emulsion reverse bupivacaine cardiotoxicity in rat by Weinberg [21] in 1998. Then, “Lipid sink” was further confirmed in the treatment of lipid emulsion on local anesthetics and other lipophilic drugs [22, 23]. Above all, we hypothesized the inflammatory mediators, oxygen radicals and lipid peroxidation products might be drawn into this “lipid sink”. This hypothesis may explain the reducing of the toxic substances concentration in plasma, and finally protect the pulmonary function in limb ischemia/reperfusion injury. However, we did not measure the extracting rate of inflammatory mediators in plasma as the explanation of “lipid sink”, and the relevant mechanism need to be further studied.

Our study had limitations: (1) we did not determine the lower limb inflammation directly, (2) the mechanism of the favorable effect of lipid emulsion on limb ischemia/reperfusion remains elusive. (3) we did not study the effect of multiple doses of lipid emulsion or the timing of their administration in regard to differing degrees of pulmonary injury. (4) spontaneous breathing in animals under general anesthesia might lead to a difficult interpretation of the CO_2_ levels when no mechanical ventilation is provided- as it is dependent on the depth of anesthesia. (5) the duration of reperfusion was fixed, therefore we could not quantify lung damage/pulmonary impairment and its relation to lipid emulsion administration at various time points during ischemia and reperfusion.

## Conclusion

Our study demonstrates that limb ischemia/reperfusion injury to the tissues leads to increased levels of plasma inflammatory mediators, plasma lipid peroxidation, and pulmonary dysfunction after limb ischemia/reperfusion, and is significantly attenuated by the administration of lipid emulsion (Intralipid, 20%). Thus, suggesting the administration of lipid emulsion is effective in combating limb ischemia/reperfusion injury by reducing lipid peroxidation and the resultant systemic inflammatory response.

## Abbreviation

ABG: Arterial blood gas
CAMs: Cell adhesion molecules
HR: Heart rate
IL-1β: Interleukin-1β
IL-6: Interleukin-6
Lb: Lamellar body
LIS: Lung injury score
MAP: Mean arterial pressure
MDA: Malondialdehyde
MIS: Muscle injury score
Mit: Mitochondria
MPO: Myeloperoxidase
N: Nucleus
PMNs: Polymorphonuclear leukocytes
PPAR-α: Peroxisome proliferator-activated receptor alpha
SOD: Superoxide dismutase
SR: Sarcoplasmic reticulum
TNF-α: Tumor necrosis factor-α
W/D: Wet/dry
