# Erratum to: Lipid emulsion mitigates impaired pulmonary function induced by limb I/R in rats through attenuation of local cellular injury and the subsequent systemic inflammatory response/inflammation

**DOI:** 10.1186/s12871-017-0407-2

**Published:** 2017-09-04

**Authors:** Fangfang Xia, Yun Xia, Sisi Chen, Lulu Chen, Weijuan Zhu, Yuanqing Chen, Thomas J. Papadimos, Xuzhong Xu, Le Liu

**Affiliations:** Department of Anesthesiology, the First Affiliated Hospital of Wenzhou, Medical University, Zhejiang, China

## Erratum

Following publication of the original article [1], the authors pointed out the title was complete, and that it should read "Lipid emulsion mitigates impaired pulmonary function induced by limb I/R in rats through attenuation of local cellular injury and the subsequent systemic inflammatory response/inflammation" instead of "Lipid emulsion mitigates impaired pulmonary function induced by limb I/R in rats through attenuation of local cellular injury and the subsequent systemic inflammatory".
